# Prospective Evaluation of Super-Resolution Ultrasound for Differentiating Benign and Malignant Small Breast Nodules

**DOI:** 10.3390/diagnostics16213549

**Published:** 2026-10-28

**Authors:** Xuan Liu, Jue Jiang, Zhixin Zhang, Yaning Chen, Xin Lu, Juan Wang, Shanshan Yu

**Affiliations:** Department of Ultrasound, The Second Affiliated Hospital, Xi’an Jiaotong University, 157 Xiwu Road, Xincheng District, Xi’an 710004, China

**Keywords:** Super-Resolution Ultrasound, cluster analysis, small breast nodules, microvascular parameters, differential diagnosis

## Abstract

**Objective:** The objectives of this study were to identify key super-resolution ultrasound (SR US) microvascular parameters via cluster analysis and evaluate their value in differentiating benign from malignant breast nodules of size ≤2 cm, and to assess the added value of integrating conventional US features. **Methods:** This study screened 481 breast nodules by conventional and SR US; 123 nodules of size ≤2 cm were included. Spearman hierarchical clustering, within-cluster principal component analysis, and iterative variance inflation factor elimination selected core parameters. With histopathology as the reference, five models were built: conventional US morphological features (M1), quantitative SR US (M2), M2 plus enlarged enhancement area and penetrating vessels (M3), Model 1 plus enlarged enhancement area and penetrating vessels (M4), and all features (M5). Performance was compared by AUCs and the DeLong test, with bootstrap optimism correction, Hosmer–Lemeshow calibration, and decision curve analysis (DCA). **Results:** Five parameters were retained: vascular density (VD), fractal dimension (FD), mean intensity (Mean Inten), standard deviation of velocity (Std Vel), and minimum velocity (Min Vel). The AUCs for M1-M5 were 0.702, 0.752, 0.820, 0.793, and 0.852, and their sensitivity, specificity, positive predictive value, and negative predictive value (%) were 50.0/82.2/65.8/70.6, 58.0/82.2/69.0/74.1, 86.0/65.8/63.2/87.3, 80.0/69.9/64.5/83.6, and 88.0/71.2/67.7/89.7, respectively; M5 performed best (AUC 0.852; sensitivity 88.0%, specificity 71.2%, PPV 67.7%, NPV 89.7%). The bootstrap-corrected AUCs were 0.684, 0.716, 0.779, 0.732 and 0.799; calibration was adequate and DCA confirmed a net benefit for SR US models. **Conclusions:** VD, FD, Mean Inten, Std Vel, and Min Vel differentiate benign from malignant breast nodules of size ≤ 2 cm. SR US outperforms conventional US, and integrating morphologic features further improves discrimination.

## 1. Introduction

Breast cancer is among the most common malignant tumors in women [1], and early, accurate diagnosis is central to improving patient prognosis. In the American Joint Committee on Cancer (AJCC) staging system, a maximum tumor diameter of 2 cm separates T1 from T2 disease [2]; T1N0M0 represents early-stage, localized breast cancer and carries the most favorable prognosis [3]. This size threshold also has pivotal therapeutic implications: lesions ≤ 2 cm in size are amenable to breast-conserving surgery [4], sentinel lymph node biopsy can replace axillary lymph node dissection to reduce morbidity [5], and most such patients can forgo adjuvant chemotherapy [6]. Consequently, early screening and diagnosis of ≤2 cm nodules are critically important for patients with breast lesions.

Conventional ultrasound has limited ability to visualize early microcirculatory perfusion of such lesions. Super-resolution ultrasound (SR US) represents a major advance in this regard. By overcoming the acoustic diffraction limit [7], SR US markedly enhances spatial resolution while clearly visualizing intratumoral microvascular architecture, providing a quantitative basis for lesion characterization.

To date, SR US has seen preliminary application in the thyroid [8], liver [9,10], prostate [11], superficial lymph nodes [12,13], and eye [14] and in perinatal ischemic stroke [15]; however, its application to small breast nodules remains exploratory [16,17]. Existing studies are constrained by two principal limitations. First, substantial information redundancy exists among the multiple microvascular parameters, and rigorous methods for parameter selection are still lacking. Second, systematic comparisons between SR US parameters and conventional ultrasound morphologic features are scarce, and studies targeting small nodules are especially lacking.

Accordingly, this study constructed a parameter dimensionality-reduction framework based on hierarchical clustering to identify key microvascular parameters, aiming to (i) determine whether these parameters outperformed morphologic assessment and (ii) evaluate whether integrating conventional ultrasound features yielded additional diagnostic benefit—thereby clarifying the optimal application of SR US for the diagnosis of small breast nodules.

## 2. Materials and Methods

### 2.1. Study Design, Data, and Image Acquisition

#### 2.1.1. Study Design and Study Population

This study was approved by the Medical Ethics Committee of the Second Affiliated Hospital of Xi’an Jiaotong University (approval No. 190/2026, approved on 4 June 2026) and was conducted in accordance with the Declaration of Helsinki; written informed consent was obtained from all participants.

**Figure 1:**
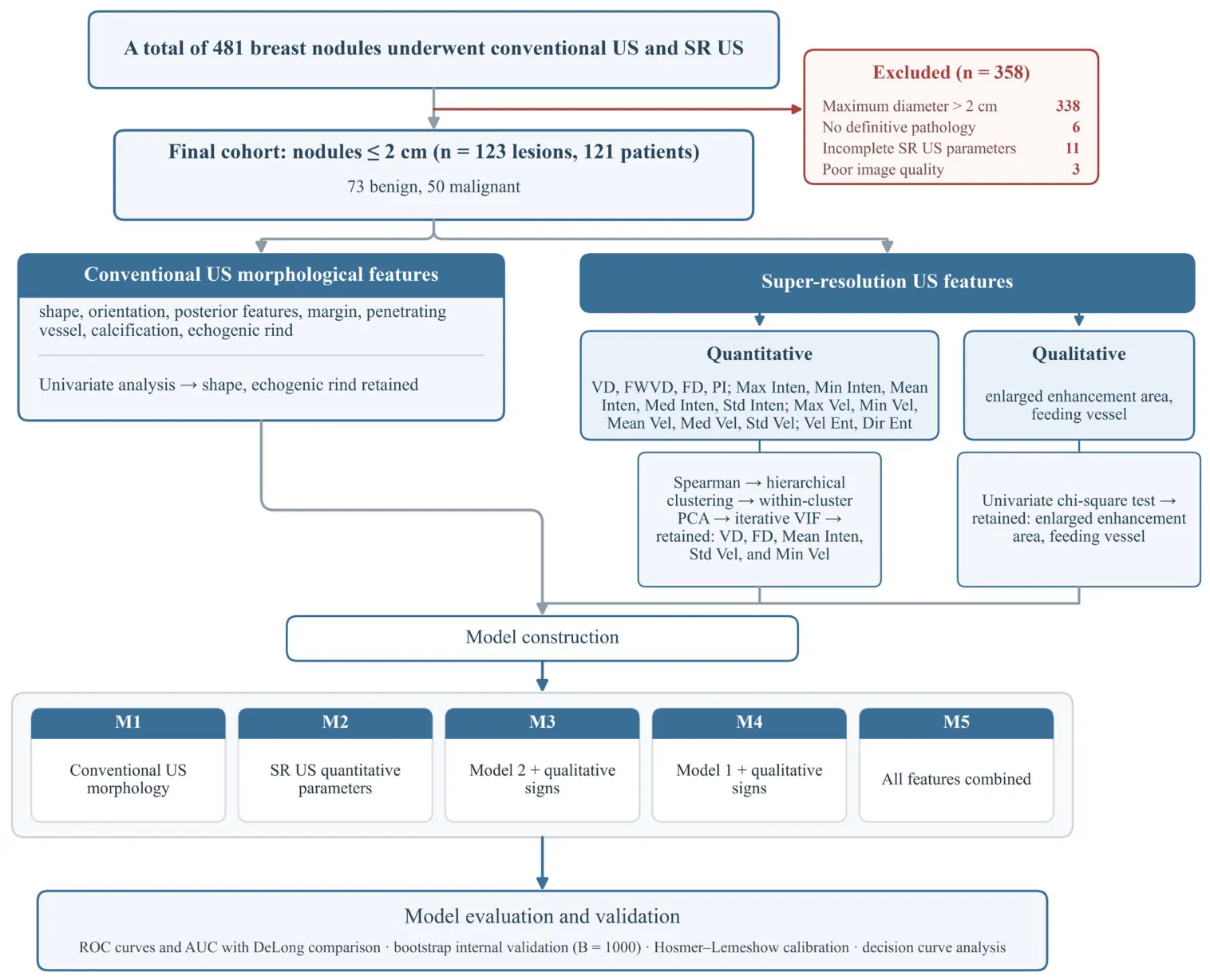
Study design and analytical pipeline.

This prospective, single-center study consecutively enrolled women with clinically or imaging-detected breast nodules who were referred to a tertiary hospital (a diagnostic, non-screening cohort). Conventional and SR US were performed for all participants, whereas mammography was obtained as part of routine clinical care when indicated and was not a standardized study procedure. To the 481 breast nodules initially assessed (Figure 1), we applied the following exclusion criteria: (1) maximum lesion diameter > 2 cm (*n* = 338); (2) no definitive histopathologic diagnosis (*n* = 6); (3) incomplete SR US parameters (*n* = 11); and (4) poor image quality (*n* = 3). A total of 123 breast nodules from 121 women (two women each had two nodules) were ultimately included, comprising 73 benign and 50 malignant lesions. The mean patient age was 48.4 ± 12.4 years (range, 20–79 years). Histopathologic results from core-needle biopsy (CNB) and surgical excision served as the reference standard, and the interpreting pathologists were blinded to the SR US findings.

#### 2.1.2. Baseline Characteristics

Malignant lesions had a significantly larger maximum diameter (*p* = 0.048) and minimum diameter (*p* = 0.004) than benign lesions, and patients with malignant lesions were significantly older (*p* < 0.001) (Table S1).

#### 2.1.3. Pathological Characteristics

In the benign group, fibroadenoma was the most common diagnosis (*n* = 30), followed by usual ductal hyperplasia (*n* = 18), intraductal papilloma (*n* = 7), inflammatory lesions (*n* = 4), fibroadenomatoid hyperplasia (*n* = 3), cystic hyperplasia (*n* = 3), adenosis (*n* = 3), fibroadenoma with adenosis (*n* = 2), cystic hyperplasia with fibroadenomatoid hyperplasia (*n* = 1), papillary neoplasm (*n* = 1), and benign phyllodes tumor (*n* = 1). The malignant group consisted predominantly of invasive ductal carcinoma (*n* = 40), with the remainder including ductal carcinoma in situ (*n* = 4), invasive lobular carcinoma (*n* = 2), intraductal papillary carcinoma with in situ carcinoma (*n* = 2), grade 1 tubular carcinoma (*n* = 1), and Paget disease with grade 2 ductal carcinoma (*n* = 1).

#### 2.1.4. Image Acquisition

Conventional ultrasound and SR US were performed using a Mindray Resona A20 system (Mindray Bio-Medical Electronics Co., Ltd., Shenzhen, China) equipped with an SL10-3U linear-array transducer (2.5–11.0 MHz). Conventional US and color Doppler flow imaging (CDFI) images were obtained first.

SonoVue (Bracco, Milan, Italy) was used as the contrast agent and was reconstituted with 5 mL of 0.9% saline (Shijiazhuang No.4 Pharmaceutical Co., Ltd., Shijiazhuang, China). Imaging was performed in a contrast-specific mode using a low mechanical index (MI, 0.08). Patients were examined in the supine position with both arms raised to fully expose the breasts and axillae. A 2.4 mL bolus of contrast agent was injected through an antecubital vein, followed by a 5 mL saline flush. Continuous cine loops were recorded for at least 90 s. Super-Resolution Imaging was initiated immediately upon microbubble arrival within the lesion and lasted 10 s.

### 2.2. Conventional US Feature Evaluation

The conventional US features evaluated included shape, orientation, posterior features, margin, calcifications, and echogenic rind, all of which were defined and categorized according to the ACR BI-RADS^®^ v2025 ultrasound lexicon [18]. A penetrating vessel was defined as a vessel coursing from outside the lesion into it on power Doppler imaging [19]. All image and cine data were independently reviewed by two sonographers with more than 5 years of experience, and discrepancies were resolved by consensus with a senior physician with more than 10 years of experience. Both readers were blinded to the histopathologic results. These seven conventional US features were visually assessed by the readers and recorded as categorical (qualitative) variables.

### 2.3. SR US Display Modes and Feature Evaluation

SR US images were displayed in four modes: vascular density image, flow velocity image, flow direction image, and flow density–direction image. No adverse events related to the ultrasound contrast agent were observed during the examinations.

From the four SR US display modes, two qualitative signs were assessed: the presence of an enlarged enhancement area and the presence of penetrating vessels. These two signs were visually assessed by the readers and recorded as binary variables (present or absent).

### 2.4. SR US Parameter Extraction

For each lesion, the reader manually delineated a region of interest (ROI) on the SR US image. The SRI post-processing software (version 7; Mindray Bio-Medical Electronics Co., Ltd., Shenzhen, China) then automatically generated all quantitative parameters offline from this ROI, including the vascular density (VD), flow-weighted vascular density (FWVD), fractal dimension (FD), and perfusion index (PI); intensity-related parameters (maximum intensity [Max Inten], minimum intensity [Min Inten], mean intensity [Mean Inten], median intensity [Med Inten], and standard deviation of intensity [Std Inten]); velocity-related parameters (maximum velocity [Max Vel], minimum velocity [Min Vel], mean velocity [Mean Vel], median velocity [Med Vel], and standard deviation of velocity [Std Vel]); and entropy-related parameters (velocity entropy [Vel Ent] and direction entropy [Dir Ent]). These parameters were treated as continuous variables. A complete list of all imaging parameters and features, with their types and value ranges, is provided in Table S2.

### 2.5. Statistical Analysis Methods

Statistical analyses were performed in R (version 4.5.2; R Foundation for Statistical Computing, Vienna, Austria) using the pROC package (version 1.19.0.1) for ROC analysis and AUC comparisons; all remaining procedures were implemented with functions written in base R. Continuous variables are expressed as the mean ± standard deviation or median according to their distribution, and between-group comparisons were performed with the Student t test or the Mann–Whitney U test. Categorical variables were compared between groups with the chi-square test or the Fisher exact test.

All conventional US features were first evaluated with univariate analysis, and features with *p* < 0.05 were selected for further analysis.

Sixteen quantitative parameters were extracted by SRI software. Min Inten was excluded because its values were close to zero, leaving 15 parameters for further analysis. An unsupervised framework was used for dimensionality reduction and parameter selection. First, because the distributions of most parameters were skewed (non-normal), a Spearman rank-correlation matrix was computed (Figure S1) and converted into a dissimilarity matrix (d = 1 − |r|), and average-linkage hierarchical clustering was performed with a cutoff at d = 0.20 (equivalent to |r| ≥ 0.80) [20]. Second, principal component analysis (PCA) was applied to the standardized variables within each cluster, and the variable with the largest absolute loading on the first principal component (PC1) was selected as the cluster representative. In this process, the original candidate variables, rather than the composite PC1 scores, were carried forward into modeling. Third, collinearity was examined with the variance inflation factor (VIF), and the variable with the highest VIF was iteratively removed until the VIF of every remaining variable was below 5 [21], a commonly adopted threshold indicating negligible multicollinearity.

The two SR US qualitative signs (enlarged enhancement area and penetrating vessels) were evaluated with univariate chi-square tests, and signs with *p* < 0.05 were retained.

With histopathologic results as the dependent variable, five multivariable binary logistic regression models were constructed by maximum likelihood using the glm function (binomial family, logit link): Model 1 (conventional US features), Model 2 (SR US quantitative parameters), Model 3 (Model 2 plus qualitative signs), Model 4 (Model 1 plus qualitative signs) and Model 5 (all features combined).

For each model, the optimal diagnostic threshold was set at the maximum Youden index. The Youden index (J = sensitivity + specificity − 1) ranges from 0 to 1, and the cutoff that maximizes J provides the optimal balance between sensitivity and specificity [22]. At this threshold, we calculated the sensitivity (SEN), specificity (SPE), positive predictive value (PPV), negative predictive value (NPV), accuracy, and Youden index. Model discrimination was compared using receiver operating characteristic (ROC) curves, the area under the ROC curve (AUC), and the DeLong test. Calibration was assessed with the Hosmer–Lemeshow test and calibration curves, net clinical benefit with decision curve analysis, and internal validation with optimism-corrected AUCs from 1000 bootstrap resamples.

Least absolute shrinkage and selection operator (LASSO) regression was not used for model construction because the limited number of events and the relatively large number of candidate parameters would make selection prone to overfitting; it was applied only as a sensitivity analysis.

## 3. Results

### 3.1. Conventional US Features for Differentiating Malignant from Benign Lesions

Univariate analysis of conventional US features showed that only shape (*p* = 0.034) and echogenic rind (*p* < 0.001) differed significantly between benign and malignant lesions (Table 1).

### 3.2. Selection of SR US Hemodynamic Parameters

**Figure 2:**
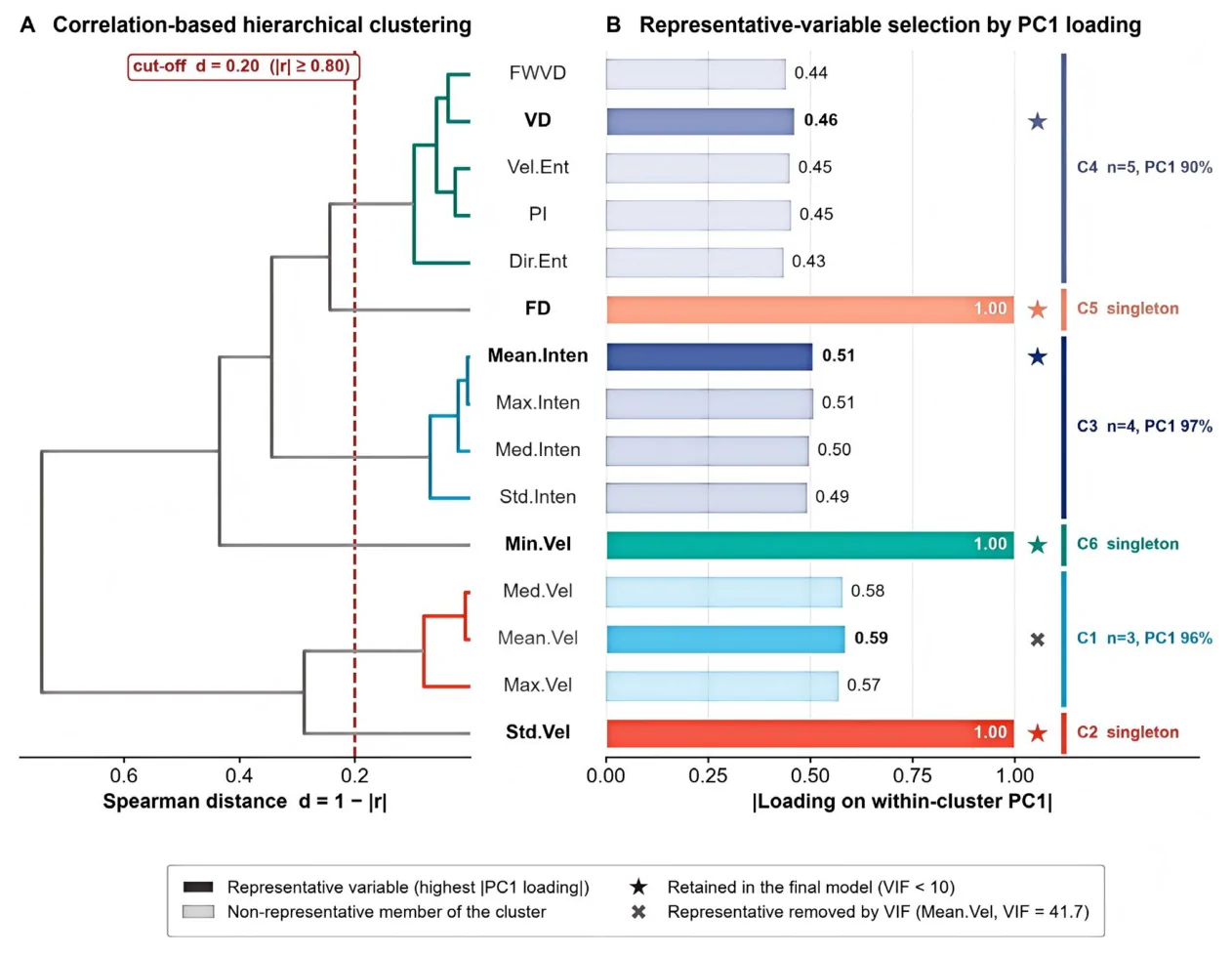
Feature selection pipeline: correlation-based hierarchical clustering of SR US parameters and representative-variable selection by first-principal-component loading.

Spearman-correlation hierarchical clustering (Figure 2) formed six clusters: a velocity cluster (Max Vel, Mean Vel, Med Vel), an intensity cluster (Max Inten, Mean Inten, Med Inten, Std Inten), a vascular density and heterogeneity cluster (VD, FWVD, PI, Vel Ent, Dir Ent), and three singletons (Std Vel, FD, Min Vel). Principal component analysis within the three multi-parameter clusters (Table S3) selected the variable with the largest absolute loading on PC1 as the representative of each cluster, explaining 96.4%, 97.0%, and 89.7% of respective within-cluster variance. Together with the three singletons, these selections yielded six candidates: Mean Vel, Mean Inten, VD, Std Vel, FD, and Min Vel. Iterative VIF elimination removed Mean Vel first (VIF = 41.7, strongest collinearity); the remaining five all had VIF < 5 (Std Vel 1.12, Mean Inten 1.97, VD 3.65, FD 2.34, Min Vel 2.12) and were retained for model construction.

### 3.3. Univariate Analysis of SR US Qualitative Signs

Both enlarged enhancement area (*p* < 0.001) and penetrating vessels (*p* = 0.034) were detected significantly more frequently in malignant than in benign lesions (Table S4).

### 3.4. Diagnostic Performance of Prediction Models

**Figure 3:**
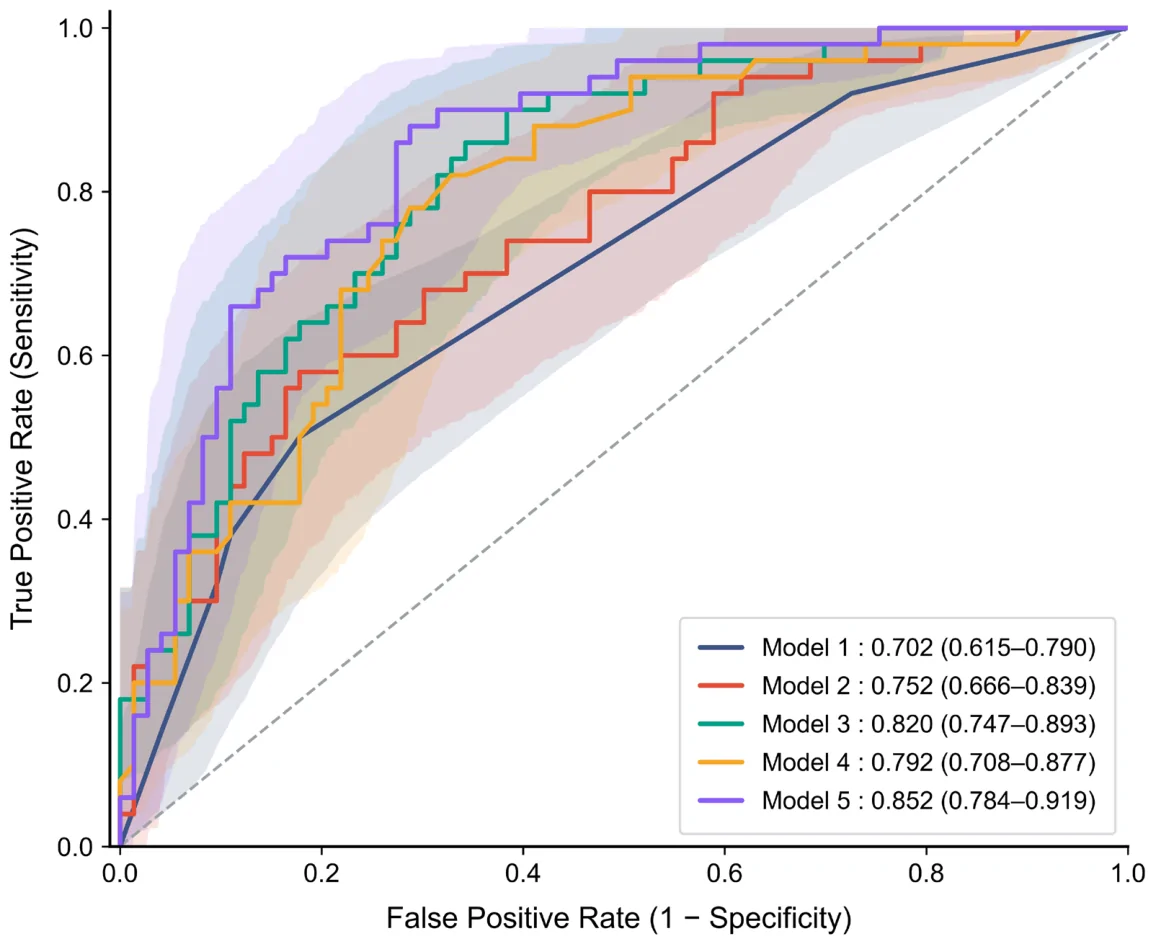
Receiver operating characteristic curves of the five prediction models. Note: Shaded areas indicate the corresponding 95% confidence intervals estimated by bootstrapping with 1000 resamples.

The conventional US model (M1) achieved an AUC of 0.702 with a sensitivity of only 50.0% and a PPV of 65.8%. The SR US quantitative model (M2) showed better discrimination (AUC = 0.752; SEN, 58.0%; PPV, 69.0%). After enlarged enhancement area and penetrating vessels were added to the quantitative parameters (M3), the AUC increased further to 0.820, with a substantial rise in sensitivity to 86.0% and a PPV of 63.2%. Model 4 (M1+ qualitative signs) yielded an AUC of 0.793 (SEN, 80.0%; PPV, 64.5%). The all-feature model (M5) achieved the highest discrimination (AUC = 0.852; SEN, 88.0%; PPV, 67.7%) (Figure 3, Table 2).

**Figure 4:**
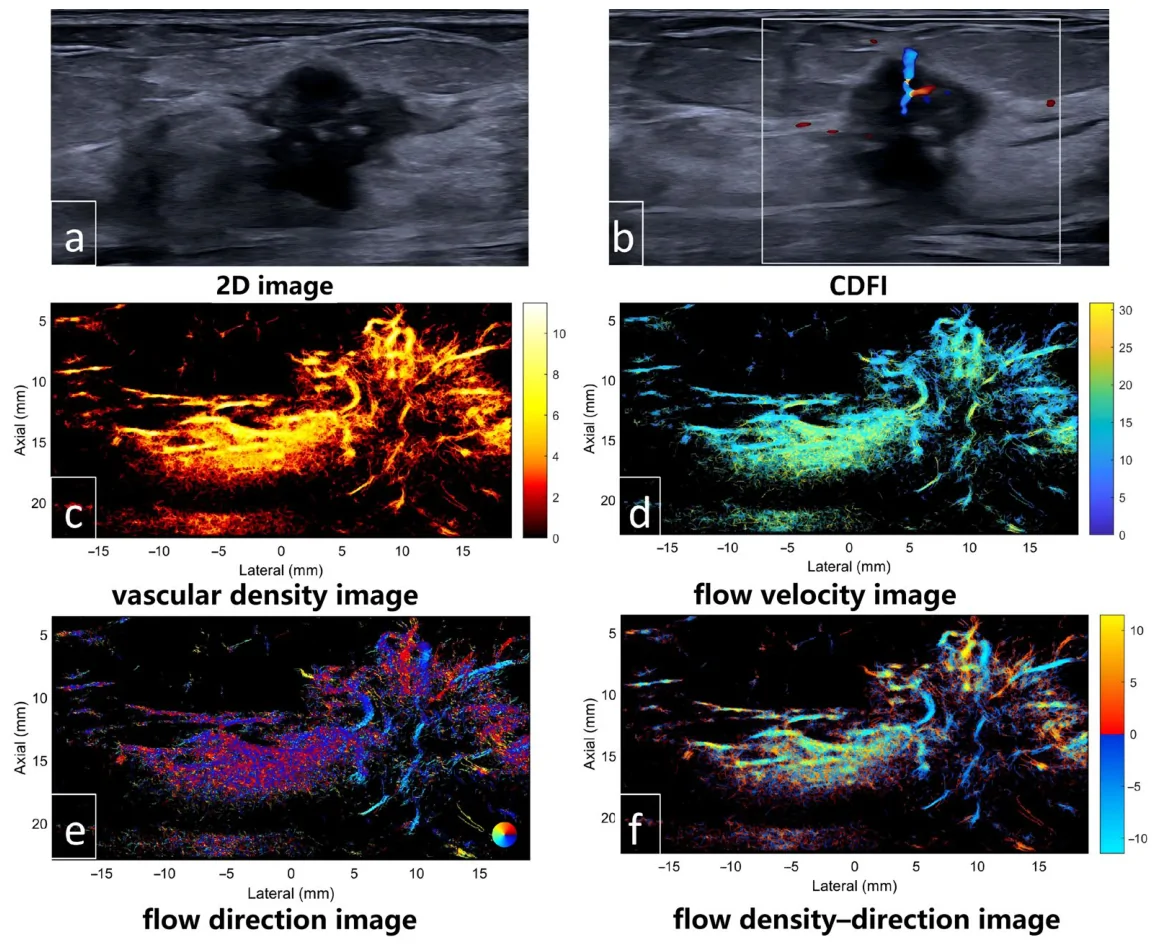
SR US images of a 53-year-old woman with invasive ductal carcinoma. (**a**) The two-dimensional image shows an irregular mass with indistinct margins, measuring approximately 14.7mm × 8.4 mm. (**b**) CDFI demonstrates linear blood flow signals. (**c**–**f**) SR US modes reveal dense, tortuous microvasculature with heterogeneous flow velocity and disorganized flow direction. Quantitative parameters: vascular density = 0.60, fractal dimension = 1.71, mean intensity = 2.41, standard deviation of velocity = 6.66, and minimum velocity = 2.10. M2–M5 yielded predicted probabilities of malignancy of 0.63, 0.70, 0.53 and 0.84, respectively, and the lesion was classified as malignant, consistent with postoperative pathology. Note: CDFI = color Doppler flow imaging; M2/M3/M4/M5 = Models 2/3/4/5.

**Figure 5:**
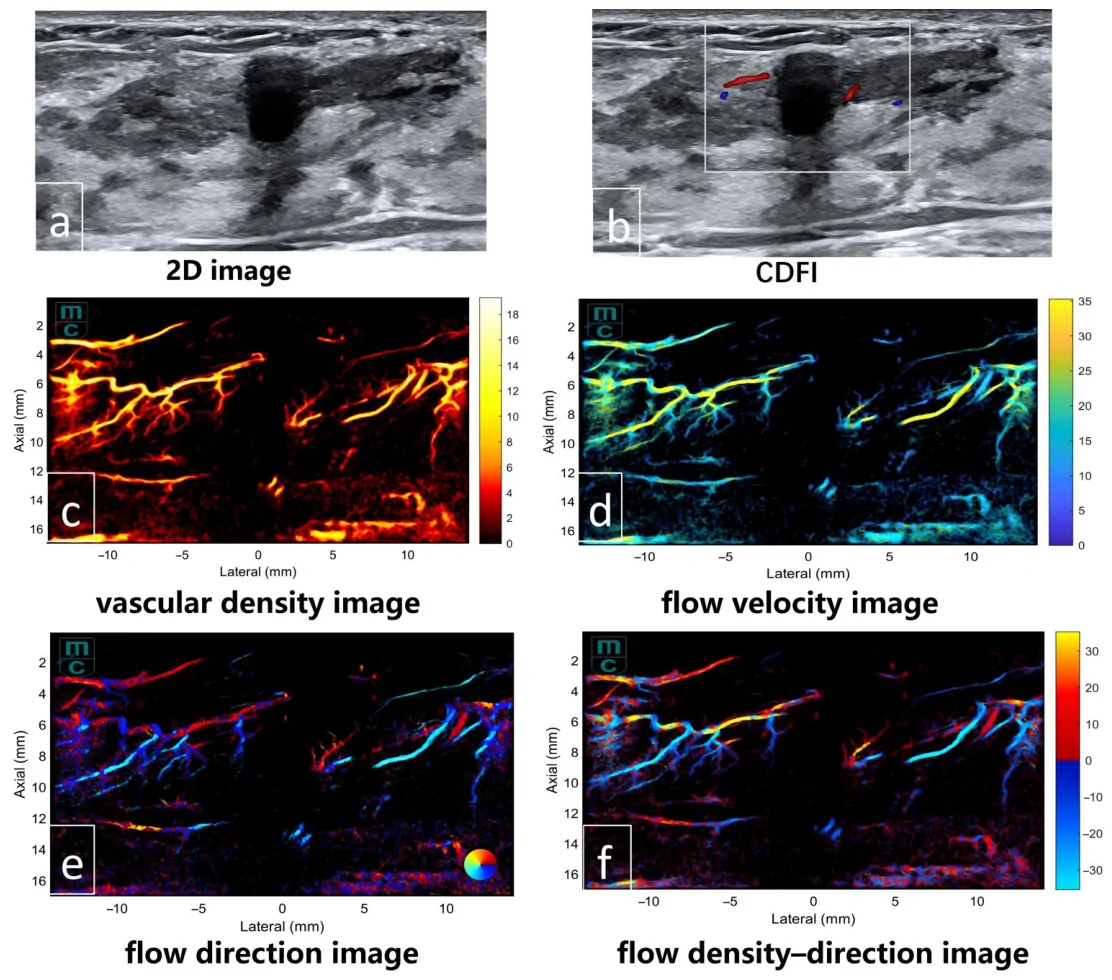
SR US images of a 44-year-old woman with fibroadenoma. (**a**) The two-dimensional image shows an oval mass with relatively clear margins, measuring approximately 6.4 mm × 3.7 mm. (**b**) CDFI demonstrates linear blood flow signals. (**c**–**f**) SR US modes demonstrate sparse, regularly distributed microvessels and homogeneous flow velocity. Quantitative parameters: vascular density = 0.21, fractal dimension = 1.11, mean intensity = 6.42, standard deviation of velocity = 10.3, and minimum velocity = 0.59. The predicted probabilities of malignancy were 0.15, 0.06, 0.127 and 0.04 for M2–M5, respectively, and the lesion was classified as benign, also consistent with pathology.

Representative SR US images of a malignant nodule and a benign nodule are shown in Figure 4 and Figure 5.

### 3.5. Model Validation and Clinical Benefit

**Figure 6:**
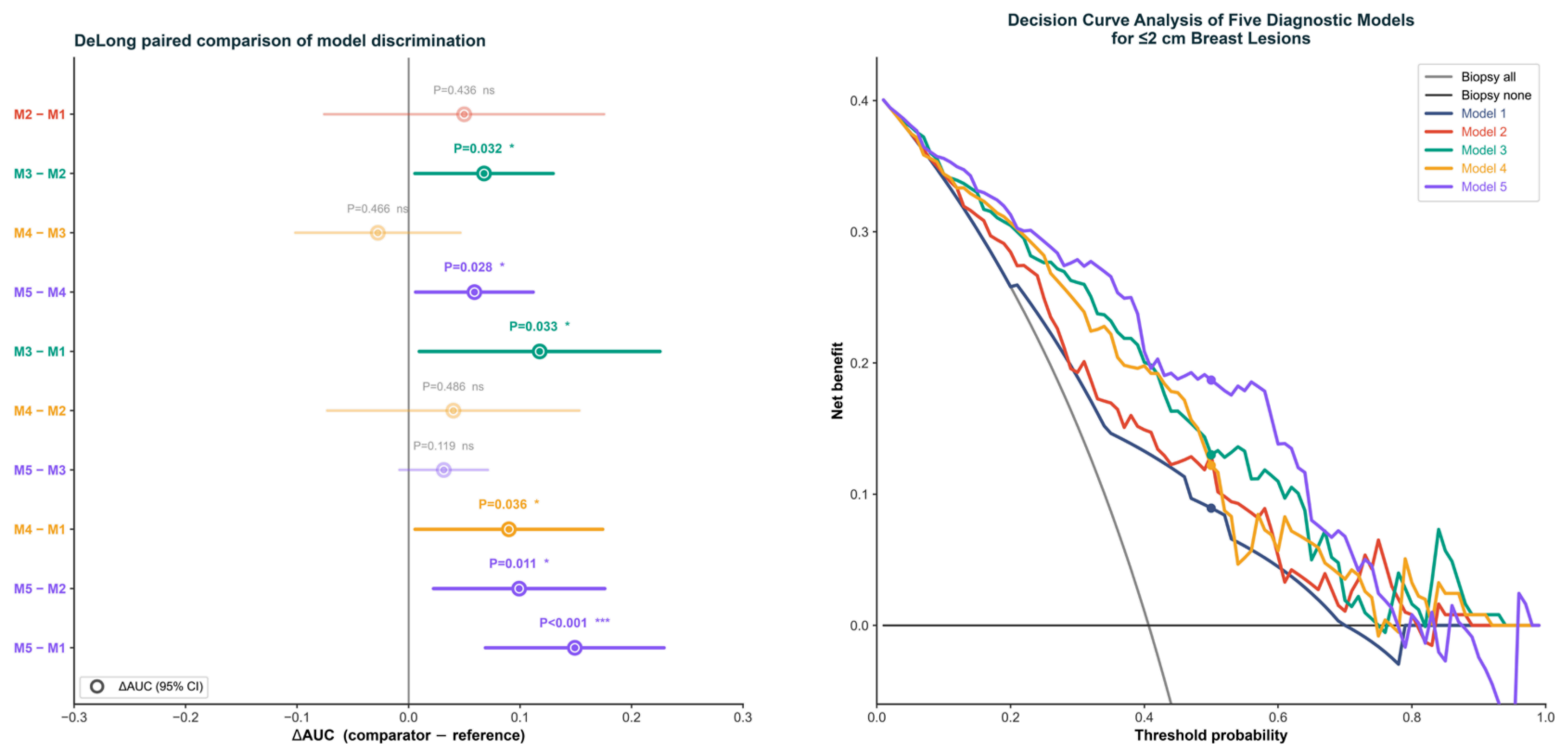
Paired model comparison and decision curve analysis. Note: (**Left**) DeLong paired comparison of model discrimination. (**Right**) Decision curve analysis shows net benefit across threshold probabilities.

The DeLong test showed that the improvement of M2 (SR US-based quantitative parameters) over M1 (conventional US features) was not statistically significant (*p* = 0.436). After enlarged enhancement area and penetrating vessels were added to the quantitative parameters, M3 (M2 plus qualitative signs) was significantly better than M2 (*p* = 0.032) and also significantly better than M1 (*p* = 0.033). M4 (conventional US morphological features plus the two qualitative signs) was not significantly different from M3 (*p* = 0.466) but was significantly better than M1 (*p* = 0.036). M5 (all-features model) was not significantly better than M3 (*p* = 0.119) but showed the greatest improvement over M1 (*p* < 0.001) (Figure 6).

**Figure 7:**
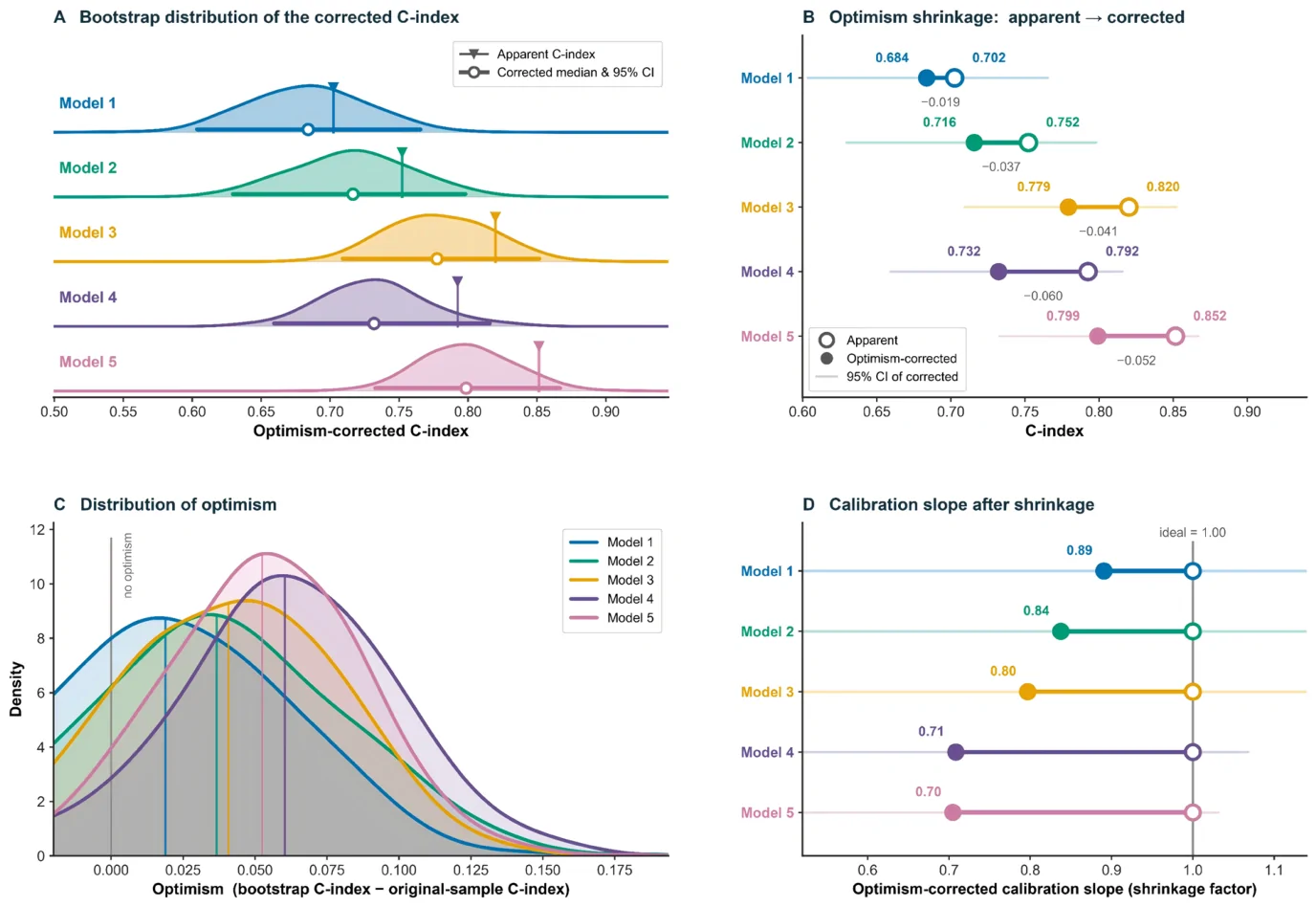
Internal validation via bootstrap optimism correction.

After bootstrap optimism correction (1000 resamples), the AUCs of the five models were 0.684, 0.716, 0.779, 0.732 and 0.799, respectively, with an optimism bias below 0.06 in all models, indicating a low risk of overfitting (Figure 7).

Regarding calibration, the Hosmer–Lemeshow test yielded *p* values of 0.780, 0.830, 0.840, 0.950 and 0.970 for the five models, indicating good agreement between predicted probabilities and observed malignancy rates (Figure S2).

Decision curve analysis showed a positive net clinical benefit for the SR US-based models across the range of threshold probabilities relevant to biopsy decisions (Figure 6).

## 4. Discussion

Conventional US has limited clinical value for characterizing breast nodules of size ≤ 2 cm. Although features such as lesion shape and echogenic rind differ significantly between benign and malignant lesions, their substantial overlap in small lesions limits diagnostic specificity. This limitation is compounded by the high operator dependence of conventional US; the discrimination achieved by junior physicians can approach the level of chance [23]. This limitation was reflected in our conventional US model (M1), which achieved an AUC of only 0.702 in this study. SR US was therefore introduced to provide objective microcirculatory information.

From the 15 quantitative parameters, cluster analysis retained five: VD, FD, Mean Inten, Std Vel, and Min Vel. VD and FD reflect the microvessel number and spatial morphology, respectively. VEGF-driven proliferation of immature neovessels elevates microvascular density [24,25,26], which relates to invasive potential and neoadjuvant chemotherapy response [27]; aberrant sprouting yields tortuous, shunt-bearing vessels [28] whose spatial disorder is captured by FD, consistent with prior SR US reports of higher vascular density (VD) [29] and fractal dimension (FD) [30] in malignancy. Mean Inten is an amplitude-based parameter subject to acoustic confounding. Among the velocity parameters, Std Vel and Min Vel showed opposing trends in the illustrative lesions (lower Std Vel and higher Min Vel in the malignant nodule), indicating a compressed, less dispersed velocity distribution. This pattern aligns with results from Li et al. [31], who found higher mean flow velocity in benign lesions, but contrasts with those from Zhang et al. [29], who reported higher microvascular flow velocity in malignant lesions. The discrepancy likely reflects differences in velocity definitions and lesion size.

To assess the robustness of our unsupervised framework, a sensitivity analysis using LASSO regression was performed. LASSO retained seven parameters: FWVD, FD, Med Inten, Std Inten, Med Vel, Std Vel, and Vel Ent (Table S5). The resulting seven-parameter model achieved an AUC of 0.781 (bootstrap optimism-corrected, 0.722) (Table S6), which was comparable to that of the five-parameter model derived from our unsupervised framework (AUC, 0.752; corrected, 0.716). These results support the robustness of our feature selection.

M2, composed of the five SR US quantitative parameters, achieved slightly better discrimination than the conventional model, M1 (AUC, 0.752 vs. 0.702; SEN, 58.0% vs. 50.0%). After bootstrap optimism correction (1000 resamples), the AUCs were 0.716 for M2 and 0.684 for M1; the optimism-corrected discrimination of M2 remained higher than that of M1, indicating that the quantitative parameters themselves carry diagnostic value.

M3, which added the two qualitative signs (enlarged enhancement area and penetrating vessels) to M2, reflected the overall diagnostic performance of SR US. Its sensitivity increased from 58.0% in M2 to 86.0%, with an AUC of 0.820. The discrimination of M3 is comparable to the previously reported diagnostic performance of conventional US [32], suggesting that SR US can attain the diagnostic performance of experienced radiologists without being constrained by operator experience.

Of note, M2 achieved specificity equal to that of M1, but with a higher PPV (69.0% vs. 65.8%) and NPV (74.1% vs. 70.6%); the specificity decreased to 65.8% after the enlarged enhancement area and penetrating vessels were incorporated into M3, accompanied by a decrease in PPV to 63.2% and an increase in NPV to 87.3%, and it rose slightly to 71.2% when morphologic features were added to M5, which yielded a PPV of 67.7% and the highest NPV among the five models (89.7%). A likely explanation for this is that the additional signs in M3 reflect aberrant vascular supply, because benign lesions may also exhibit apparently disorganized vascularity [33]; when the model weights these signs heavily, highly vascularized benign lesions tend to be classified as malignant, lowering the specificity. In M5, the added morphologic features reflect the stromal response induced by invasive growth [34], a process that is relatively uncommon in benign lesions, allowing some benign lesions to be correctly reclassified and the specificity to recover.

M4 (morphology plus qualitative signs) achieved an AUC of 0.793 (SEN, 80.0%; SPE, 69.9%; PPV, 64.5%; NPV, 83.6%); this decreased to 0.732 after optimism correction, close to the value for M2 (0.716) and below that of M3 (0.779). Thus, SR US provides useful microvascular information, but its quantitative and qualitative components, when used separately, yield only limited incremental gain over conventional US features.

With the addition of morphologic features, M5 achieved the highest performance among the five models (AUC, 0.852; SEN, 88.0%; SPE 71.2%; PPV, 67.7%; NPV, 89.7%). However, the improvement in the AUC of M5 over that of M3 (AUC, 0.820; SEN, 86.0%; SPE, 65.8%; PPV, 63.2%; NPV, 87.3%) was not statistically significant (DeLong *p* = 0.119), likely attributable to limited statistical power and information overlap, suggesting that conventional morphologic features may still confer additional benefit in discrimination.

This study has several limitations. First, the single-center design and limited sample size constrain the generalizability of the conclusions, which warrant validation in multicenter prospective cohorts. Second, although most malignant lesions in this cohort corresponded to early-stage T1N0M0 disease, subgroup analyses according to lymph node status or molecular subtype were not performed; further studies may address this issue. Third, the quantitative parameters in this study merely reflected the statistical distribution of pixel intensity and flow velocity values and did not incorporate image-content-based radiomic features; future work may extend this framework by integrating texture, morphological, and channel-specific analyses.

## 5. Conclusions

VD, FD, Mean Inten, Std Vel, and Min Vel are important parameters for differentiating benign from malignant small breast nodules. SR US outperforms conventional US, and integrating morphologic features further improves discrimination.

## Figures and Tables

**Table 1:** Baseline conventional ultrasound characteristics of benign and malignant breast lesions of size ≤ 2 cm.

Characteristic	Benign (*n* = 73)	Malignant (*n* = 50)	Total (*n* = 123)	χ^2^	*p*
Shape				6.775	0.034 *
Round	8 (11.0%)	13 (26.0%)	21 (17.1%)		
Oval	21 (28.8%)	7 (14.0%)	28 (22.8%)		
Irregular	44 (60.3%)	30 (60.0%)	74 (60.2%)		
Orientation				0.098	0.754
Parallel	64 (87.7%)	42 (84.0%)	106 (86.2%)		
Non-parallel	9 (12.3%)	8 (16.0%)	17 (13.8%)		
Posterior features				0.841	0.657
No posterior features	58 (79.5%)	39 (78.0%)	97 (78.9%)		
Enhancement	2 (2.7%)	3 (6.0%)	5 (4.1%)		
Shadowing	13 (17.8%)	8 (16.0%)	21 (17.1%)		
Margin				0.088	0.766
Non-circumscribed	54 (74.0%)	39 (78.0%)	93 (75.6%)		
Circumscribed	19 (26.0%)	11 (22.0%)	30 (24.4%)		
Penetrating vessels				0.382	0.537
Absent	49 (67.1%)	30 (60.0%)	79 (64.2%)		
Present	24 (32.9%)	20 (40.0%)	44 (35.8%)		
Calcifications				1.140	0.566
Absent	53 (72.6%)	36 (72.0%)	89 (72.4%)		
Microcalcifications	14 (19.2%)	12 (24.0%)	26 (21.1%)		
Macrocalcifications	6 (8.2%)	2 (4.0%)	8 (6.5%)		
Echogenic rind				11.136	<0.001 ***
Absent	65 (89.0%)	31 (62.0%)	96 (78.1%)		
Present	8 (11.0%)	19 (38.0%)	27 (22.0%)		

Note: * *p* < 0.05; *** *p* < 0.001. χ^2^ = chi-square test.

**Table 2:** Diagnostic performance of the five prediction models.

Parameter	Model 1	Model 2	Model 3	Model 4	Model 5
	Conventional US	SR US quantitative	Model 2 + qualitative	Model 1 + qualitative	All features
AUC (95% CI)	0.702 (0.615–0.790)	0.752 (0.666–0.839)	0.820 (0.747–0.893)	0.793 (0.708–0.877)	0.852 (0.784–0.919)
Cut-off value	0.465	0.497	0.339	0.446	0.352
SEN % (95% CI)	50.0 (36.6–63.4)	58.0 (44.2–70.6)	86.0 (73.8–93.0)	80.0 (67.0–88.8)	88.0 (76.2–94.4)
SPE % (95% CI)	82.2 (71.9–89.3)	82.2 (71.9–89.3)	65.8 (54.3–75.6)	69.9 (58.6–79.2)	71.2 (60.0–80.3)
ACC % (95% CI)	69.1 (60.5–76.6)	72.4 (63.9–79.5)	74.0 (65.6–80.9)	74.0 (65.6–80.9)	78.0 (69.9–84.5)
PPV % (95% CI)	65.8 (49.9–78.8)	69.0 (54.0–80.9)	63.2 (51.4–73.7)	64.5 (52.1–75.3)	67.7 (55.6–77.8)
NPV % (95% CI)	70.6 (60.2–79.2)	74.1 (63.6–82.4)	87.3 (76.0–93.7)	83.6 (72.4–90.8)	89.7 (79.2–95.2)
Youden index	0.322	0.402	0.518	0.499	0.592

Note: Model 1 = conventional ultrasound (shape and echogenic rind); Model 2 = SR US quantitative parameters (standard deviation of velocity, mean intensity, vascular density, fractal dimension, minimum velocity); Model 3 = Model 2 plus enlarged enhancement area and penetrating vessels; Model 4 = Model 1 plus enlarged enhancement area and penetrating vessels; Model 5 = all features combined. AUC = area under the receiver operating characteristic curve; SEN = sensitivity; SPE = specificity; ACC = accuracy; PPV = positive predictive value; NPV = negative predictive value; CI = confidence interval.

## Data Availability

The data presented in this study are available on request from the corresponding author due to privacy restrictions.

## Supplementary Materials

The following supporting information can be downloaded at https://www.mdpi.com/article/10.3390/diagnostics16213549/s1, Figure S1, Pairwise Spearman rank-correlation matrix and hierarchical clustering of the 15 quantitative SR US parameters; Figure S2, Calibration curves of the five prediction models (M1–M5); Table S1, Univariate comparison of baseline characteristics between benign and malignant lesions; Table S2, Imaging parameters and features assessed by conventional US and SR US; Table S3, Principal component analysis within each cluster: PC1 variance and representative variable selection; Table S4, Univariate association of the two qualitative SR US enhancement signs with malignancy; Table S5, LASSO regression selected predictors and coefficients (lambda.min); Table S6, Performance metrics of the LASSO sensitivity-analysis model.

## Abbreviations

SR US	super-resolution ultrasound
PCA	principal component analysis
VIF	variance inflation factor
ROC	receiver operating characteristic
AUC	area under the curve
DCA	decision curve analysis
VD	vascular density
FD	fractal dimension
Mean Inten	mean intensity
Std Vel	standard deviation of velocity
Min Vel	minimum velocity
CDFI	color Doppler flow imaging
CNB	core-needle biopsy
PPV	positive predictive value
NPV	negative predictive value
LASSO	least absolute shrinkage and selection operator
CI	confidence interval
Dir Ent	direction entropy
FWVD	flow-weighted vascular density
Max Inten	maximum intensity
Max Vel	maximum velocity
Med Inten	median intensity
Med Vel	median velocity
Mean Vel	mean velocity
MI	mechanical index
PI	perfusion index
Std Inten	standard deviation of intensity
Vel Ent	velocity entropy
