# Supplementary material for: Prospective Evaluation of Super-Resolution Ultrasound for Differentiating Benign and Malignant Small Breast Nodules
Source: Diagnostics (Basel). 2026 Oct 28;16(21):3549. doi: 10.3390/diagnostics16213549 (PMC13761861; doi:10.3390/diagnostics16213549)

Supplementary Materials: Prospective Evaluation of Super Resolution Ultrasound for Differentiating Benign and Malignant Small Breast Nodules

Table S1. Univariate comparison of baseline characteristics between benign and malignant lesions.

Variable	Benign	Malignant	*p*
Maximum diameter (mm)	12.5 ± 4.5	14.2 ± 4.4	0.048*
Minimum diameter (mm)	7.7 [5.6–8.9]	9.1 [7.0–10.9]	0.004**
Age (years)	43.7 ± 10.8	55.2 ± 11.5	<0.001***

Note: * $p < 0.05$; ** $p < 0.01$; *** $p < 0.001$

Table S2. Imaging parameters and features assessed by conventional US and SR US, with parameter type and value ranges.

Parameter / Feature	Modality	Source	Type	Possible values / Range
Shape	Conventional US	Reader	Categorical	Round, oval, irregular
Orientation	Conventional US	Reader	Categorical	Parallel, Non-parallel
Posterior features	Conventional US	Reader	Categorical	No posterior features, Enhancement,

				Shadowing
Margin	Conventional US	Reader	Categorical	Circumscribed, Non-circumscribed
Penetrating vessel	Conventional US	Reader	Categorical	Absent, present
Calcifications	Conventional US	Reader	Categorical	Absent, Microcalcifications, Macrocalcifications
Echogenic rind	Conventional US	Reader	Categorical	Absent, present
VD	SR US	Software	Continuous	0.014–0.897
FWVD	SR US	Software	Continuous	0.015–5.464
FD	SR US	Software	Continuous	0.605–1.309
PI	SR US	Software	Continuous	0.210–19.045
Max Inten	SR US	Software	Continuous	1.462–13.032
Min Inten	SR US	Software	Continuous	0.00–2.30
Mean Inten	SR US	Software	Continuous	1.036–6.903
Med Inten	SR US	Software	Continuous	0.860–5.850
Std Inten	SR US	Software	Continuous	0.540–4.256

Max Vel	SR US	Software	Continuous	18.888–69.633
Min Vel	SR US	Software	Continuous	0.002–8.323
Mean Vel	SR US	Software	Continuous	9.115–33.302
Med Vel	SR US	Software	Continuous	8.408–29.155
Std Vel	SR US	Software	Continuous	4.986–18.536
Vel Ent	SR US	Software	Continuous	0.112–1.393
Dir Ent	SR US	Software	Continuous	0.124–0.964
Enlarged enhancement area	SR US	Reader	Categorical (binary)	Absent, present
Penetrating vessels (SR US sign)	SR US	Reader	Categorical (binary)	Absent, present

Note: Conventional US features were visually assessed by readers and recorded as categorical variables; SR US quantitative parameters were automatically generated by SRI software and treated as continuous variables; the two SR US qualitative signs were reader-assessed binary variables. Ranges are observed minimum–maximum values across the enrolled small-lesion cohort. Min Inten was excluded from modeling because its values approximated zero (see Methods). Intensity parameters are non-negative; the minimum intensity (Min Inten) floor is 0.

Table S3. Principal component analysis within each cluster: PC1 variance and representative variable selection.

Cluster	PC1 var.	Representative	Per-variable |PC1 loading|
C1	96.4%	Mean Vel	Max Vel=0.568; Mean Vel=0.586; Med Vel=0.578
C2	100.0%	Std Vel	Std Vel=1.000
C3	97.0%	Mean Inten	Max Inten=0.506; Mean Inten=0.507; Med Inten=0.496; Std Inten=0.490
C4	89.7%	VD	VD=0.463; FWVD=0.439; PI=0.452; Vel Ent=0.448; Dir Ent=0.433
C5	100.0%	FD	FD=1.000
C6	100.0%	Min Vel	Min Vel=1.000

Note: VD = vascular density; FWVD = flow-weighted vascular density; FD = fractal dimension; PI = perfusion index; Max Inten = maximum intensity; Mean Inten = mean intensity; Med Inten = median intensity; Std Inten = standard deviation of intensity; Max Vel = maximum velocity; Mean Vel = mean velocity; Med Vel = median velocity; Std Vel = standard deviation of velocity; Min Vel = minimum velocity; Vel Ent = velocity entropy; Dir Ent = direction entropy.

Table S4. Univariate association of the two qualitative SR US enhancement signs with malignancy.

Characteristic	Benign (n = 73)	Malignant (n = 50)	Total (n = 123)	χ^2	***p***
Enlarged enhancement area				20.199	<0.001***

Characteristic	Benign (n = 73)	Malignant (n = 50)	Total (n = 123)	χ^2	***p***
Absent	39 (53.4)	6 (12.0)	45 (36.6)		
Present	34 (46.6)	44 (88.0)	78 (63.4)		
Penetrating vessels				4.470	0.034*
Absent	46 (63.0)	21 (42.0)	67 (54.5)		
Present	27 (37.0)	29 (58.0)	56 (45.5)		

Note: * $p < 0.05$; ** $p < 0.01$; *** $p < 0.001$

Table S5. LASSO regression selected predictors and coefficients (lambda.min).

**Predictor**	**β (coefficient)**	**exp(β)**
Intercept	-4.856	0.0078
FWVD	-0.690	0.5016
FD	3.931	50.904
Med Inten	1.702	5.485
Std Inten	-2.244	0.1062
Med Vel	-0.181	0.8345
Std Vel	-0.526	0.5911
Vel Ent	2.707	14.993

Note: FWVD = flow-weighted vascular density; FD = fractal dimension; Med Inten = median intensity; Std Inten = standard deviation of intensity; Med Vel = median velocity; Std Vel = standard deviation of velocity; Vel Ent = velocity entropy.

Table S6. Performance metrics of the LASSO sensitivity-analysis model.

Metric	Value
LASSO penalty (C, 10-fold CV)	2.5595
Apparent AUC (95% CI)	0.781 (0.698–0.856)
Sensitivity	0.600
Specificity	0.822
Youden index	0.422
Bootstrap-corrected AUC	0.722

Note: AUC = area under the receiver operating characteristic curve; CV = cross-validation; CI = confidence interval.

Figure S1. Pairwise Spearman rank correlation matrix and hierarchical clustering of the 15 quantitative SR US parameters.

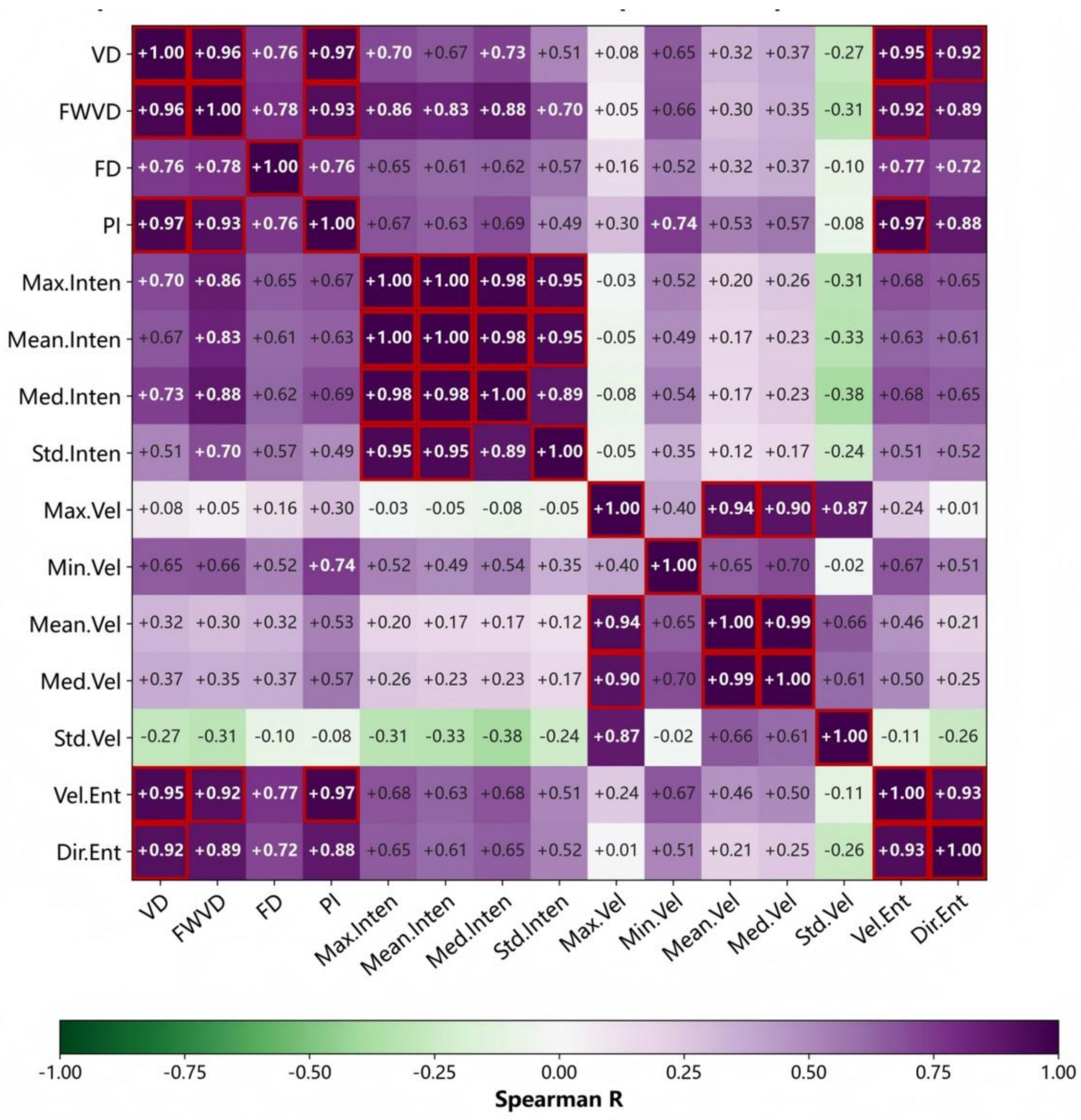

Figure S2. Calibration curves of the five prediction models (M1–M5).

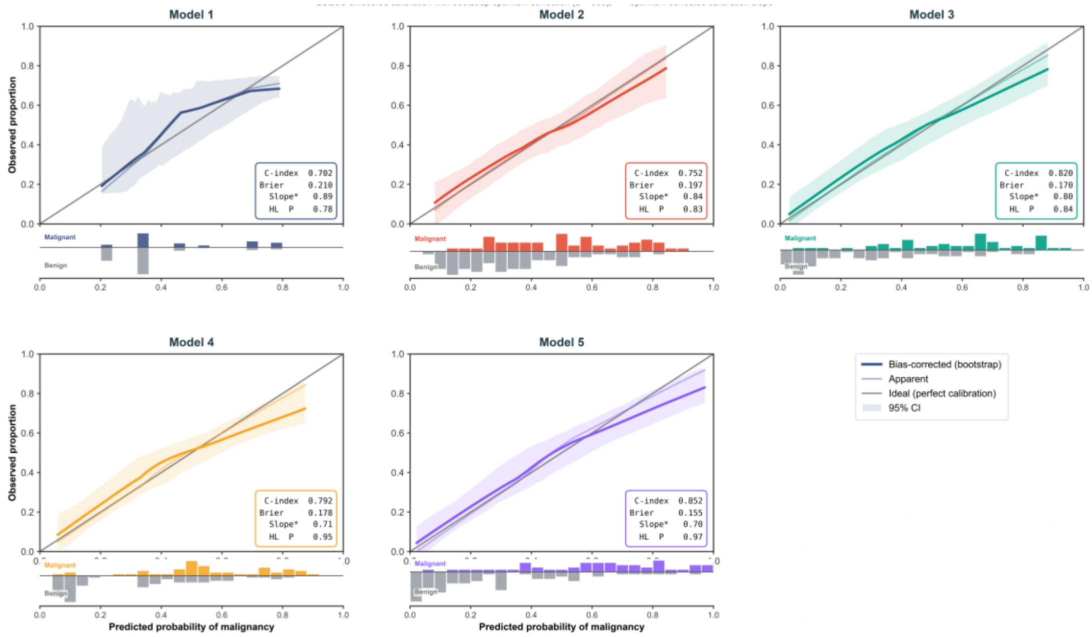

Supplement: Supplementary file 1 [file diagnostics-16-03549-s001.zip › diagnostics-4624297-supplementary.pdf]
